# *Ab Initio* Identification of Novel Regulatory Elements in the Genome of *Trypanosoma brucei* by Bayesian Inference on Sequence Segmentation

**DOI:** 10.1371/journal.pone.0025666

**Published:** 2011-10-03

**Authors:** Steven Kelly, Bill Wickstead, Philip K. Maini, Keith Gull

**Affiliations:** 1 Department of Plant Sciences, University of Oxford, Oxford, United Kingdom; 2 Centre for Mathematical Biology, Mathematical Institute, University of Oxford, Oxford, United Kingdom; 3 Oxford Centre for Interactive Systems Biology, Department of Biochemistry, University of Oxford, Oxford, United Kingdom; 4 Sir William Dunn School of Pathology, University of Oxford, Oxford, United Kingdom; 5 Centre for Genetics and Genomics, The University of Nottingham, Queen's Medical Centre, Nottingham, United Kingdom; University of Minnesota, United States of America

## Abstract

**Background:**

The rapid increase in the availability of genome information has created considerable demand for both comparative and ab initio predictive bioinformatic analyses. The biology laid bare in the genomes of many organisms is often novel, presenting new challenges for bioinformatic interrogation. A paradigm for this is the collected genomes of the kinetoplastid parasites, a group which includes Trypanosoma brucei the causative agent of human African trypanosomiasis. These genomes, though outwardly simple in organisation and gene content, have historically challenged many theories for gene expression regulation in eukaryotes.

**Methodology/Principle Findings:**

Here we utilise a Bayesian approach to identify local changes in nucleotide composition in the genome of T. brucei. We show that there are several elements which are found at the starts and ends of multicopy gene arrays and that there are compositional elements that are common to all intergenic regions. We also show that there is a composition-inversion element that occurs at the position of the trans-splice site.

**Conclusions/Significance:**

The nature of the elements discovered reinforces the hypothesis that context dependant RNA secondary structure has an important influence on gene expression regulation in Trypanosoma brucei.

## Introduction

*Ab initio* discovery of regulatory information encoded in genomic sequence is one of the most interesting and difficult challenges facing post-genome era biologists. At the heart of sequence-dependent expression-regulation is both the sequence of the gene and its encompassing genomic environment. In most eukaryotes, the first stage of sequence-dependent gene expression regulation occurs at the point of transcription initiation. This regulation is dependent on short specific sequences which can act positively or negatively to effect transcriptional regulation, and in most cases mediate their effects through the recruitment of specific DNA binding proteins. For the purposes of this work, we consider this group of short regulatory elements to encompass all transcription factor binding sites and promoter/polymerase recruitment sites. Short specific sequences also play a role in regulation of gene expression post transcription, and in this context can appear in the form of polyadenylation signals, splice sites (e.g. sequences which mark intron-exon boundaries [1]) or sites which recruit RNA binding proteins. Post-transcriptional processing is also dependent on longer sequences which function to impart structure to the emerging or mature RNA transcript, for example the hairpin loop found in the 3′ un-translated region of histone mRNAs [2]. Finally, at the point of translation, the sequence of the gene itself can play a regulatory role in its expression through its intrinsic codon bias: i.e. by using codons which correspond to different iso-accepting tRNAs with different relative abundances in the cell, translational efficiency and hence gene expression efficiency can be affected [3], [4]. Regulation beyond this point is assumed to be independent of the sequence encompassed in the gene or its genomic location.

The trypanosomatids are a monophyletic sub-group of excavate parasites [5], [6] which collectively exhibit an extraordinarily broad host-range which extends from crocodiles [7] to coconut palms [8]. Several members of the trypanosomatid group cause globally important parasitic diseases of humans including sleeping sickness, Chagas disease and leishmaniases which together kill and debilitate hundreds of thousands of people worldwide each year (http://www.who.int). The study of gene-expression in trypanosomatids has been highly productive and has led to the discovery of several fundamental aspects of transcription, some of which have subsequently been found to be utilised in many other eukaryotes. These discoveries include RNA editing [9], trans-splicing [10], [11], [12] and RNA polymerase I transcription of protein coding genes [13], [14]. Despite these early breakthroughs in understanding gene expression in trypanosomatids, we know little about the identity and role of the many regulatory elements which must govern this system. This is particularly pertinent as most protein coding genes in trypanosomatids are expressed in polycistronic transcription units where promoters appear to be absent from the 5′ end of genes. There are a few exceptions to this rule, and these include the characterisation of a number of Pol I and Pol II promoters. The characterised Pol I promoters drive expression of the ribosomal RNA and the major surface protein genes of the parasite [15], [16], [17], [18], [19], and the characterised Pol II promoter drives expression of the spliced-leader RNA [20]. While little is known about other regulatory elements which govern this system, a number of cis-elements and trans-acting protein factors have been identified [21], [22], [23], [24], [25].

In trypanosomatids, transcription of all protein coding genes by RNA polymerase II is polycistronic [26], [27], [28], [29]. The average polycistron contains ∼70 genes [30] and each gene must be processed to monocistronic RNA units before nuclear export and translation can occur. This obligatory transition to monocistronic RNA molecules is mediated by trans-splicing, a process in which a 35 nucleotide capped RNA molecule is added upstream of the start codon of each coding sequence [11], [12], [31]. Prior to the release of the trypanosomatid genomes [30], [32], [33], analysis of the few available gene sequences resulted in the proposal of a canonical trans-splicing signal. This signal was believed to be composed of four elements: 1) a branch-point adenine site (YNYURAC where Y = C or U; R = G or A, and N = any base), 2) a poly-pyrimidine tract, 3) a short variable spacer region and 4) a splice acceptor site (AG) [34]. The importance of the poly-pyrimidine tract has since been repeatedly documented [35], [36], [37], [38], however its role in the trans-splicing process or its properties have yet to be defined. Moreover, it became apparent on release of the genome sequences that one could not use these elements to locate *bona fide* trans-splice sites.

In the absence of obvious motifs controlling trans-splice site specification, two statistical methods for identifying putative trans-splice acceptor sites in the 5′ intergenic region of coding sequences were developed [39], [40], [41]. One method is based on the observation that the AG di-nucleotide that is often used as the splice acceptor site is situated in a region of DNA otherwise poor in AG di-nucleotides. The other is based on the position of an AG di-nucleotide relative to a semi-defined polypyrimidine tract element. In both studies, short specific sequences could not be identified, rather statistical approaches were taken to produce scores for individual AG sites outside of open reading frames. The convergence of both studies on an absence of “motifs” implies that secondary structure plays an important role in splice-site delineation. Moreover, discrepancies between prediction methods implies that there are extra levels of information encoded in the genome which have so far evaded characterisation.

Unlike in most eukaryotes, polyadenylation of transcripts in trypanosomatids is dependent on post-transcriptional processing of the downstream gene. It was shown in experiments with permeabilised cells that trans-splicing of a gene occurs before the polyadenylation of its upstream neighbour and that inhibition of trans-splicing leads to coupled abolition of polyadenylation [35]. Given the gene-dense nature of the trypanosomatid directional gene clusters, and the fact that trans-splicing by its nature cleaves the transcript near to the 3′ end of the preceding gene, it is easy to hypothesise that polyadenylation in trypanosomatids became dependent on trans-splicing for production of the free 3′ end. Since it appears that only one cleavage event occurs [35], and that transcripts are polyadenylated at specific reproducible sites (though often more than one site per gene [37], [42], [43] the 3′end must be degraded back to an as yet elusive polyadenylation signal. Implicit in this coupled mechanism is that there must be trans-splice sites not associated with coding sequences located downstream of genes at the ends of polycistrons. Indeed, trans-splicing to AG sites lacking any associated coding sequencing has been observed [37]. However, the role of these trans-spliced non-coding RNA sequences, apart from generating free 3′ ends, has yet to be determined.

Several methods have been developed to identify regulatory motifs in DNA sequences. The methods are diverse and can range from information-rich methods, which utilise groups of co-regulated or functionally related genes to search for regulatory sequences in upstream regions (for example [44], [45]), to information-poor methods, which simply look for over-represented sequence motifs in a collection of sequences (for example [46], [47]). To date these and other methods have failed to shed light on any recurring regulatory motifs in trypanosomatid DNA sequences. However, regulatory elements must exist, as both cell-cycle and life-cycle dependent regulation of gene expression has been extensively documented.

Given that complex patterns of gene expression regulation exist, that cis-acting regulatory motifs appear to be rare and that local nucleotide composition has been shown to be a useful marker in predicting trans-splice sites in kinetoplastid DNA sequences [40], [41]. We attempted to determine whether any common or recurring compositional elements could be identified which correlated with known RNA processing sites or which could yield insight into the how gene expression regulation is being mediated in trypanosomatid genomes. To this end we utilised a Bayesian segmentation method [48] to interrogate the fluctuations in compositional bias in trypanosomatid DNA sequences.

## Materials and Methods

### Bayesian segmentation

The Bayesian segmentation method used in this analysis is based on schema adapted from [48], [49]. In brief, the goal of segmentation is to separate out homogeneous regions from sequential data. As such, segmentation plays a critical role in data mining for a wide range of scientific and commercial/industrial applications. A holy grail of data segmentation is to discover homogeneous regions without the requirement for prior assumptions or user derived parameters. Given that DNA is composed of only four nucleotides, it presents an ideal candidate for data segmentation and homogeneous region discovery. The full description of the mathematics is provided in file S1. The Perl source code for segmenting FASTA format sequences is provided as file S2.

Our method has two prior assumptions. The first is the conceptual basis for our analysis i.e. that some regulatory elements in the genome may be defined by their local nucleotide composition. The second assumption is that we assign equal weighting to all nucleotides when identifying changes in local nucleotide composition. Given these two assumptions we use a Bayesian method to assign a probability of there being a change in nucleotide composition at every point along a given sequence. Using Bayes rule it is possible to determine the most likely number of change-points in any given sequence (File S1). Hence, for all tests the most probable number of change-points (N) given the sequence was determined and then the N most probable change points were selected as the N highest peaks. Peak selection was made on smoothed probability density profiles which were smoothed using a Gaussian kernel with σ = 3 nucleotides. We then use these inferred change-points in sequence composition to demark the edges of regions containing homogeneous local nucleotide composition. Putative regulatory elements are then identified by comparing these regions and their edges across multiple different sequences.

### Evaluation of segmentation performance

To demonstrate the ability of the method to detect changes in nucleotide composition we tested the ability of the method to recover true changes in sequence composition from simulated sequence data. In all cases the performance of the method was evaluated in terms of precision and recall.
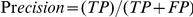


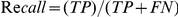
Where the number of true positives (TP) is the number of change points identified by the segmentation method that were present in the simulated sequence. The number of false positives (FP) is the number of change points identified by the segmentation method which are not present in the simulated sequence. The number of false negatives (FN) is the number of change points present in the simulated sequence not identified by the segmentation method.

### Extraction and segmentation of genome data

To determine whether known sequence features correlated with the position of inferred change points in nucleotide composition we extracted genome sequence containing known features from *Trypanosoma brucei*. We selected 5,121 intergenic regions which contained at least one annotated splice acceptor site (n = 6,577) and at least one annotated polyadenylation site (n = 5,650) [50]. In this high-throughput sequence data putative polyadenylation sites were identified as a run of 8 or more A nucleotides in the sequenced RNA molecule. As some of these sites could be false positives arising from A nucleotides occurring at the same place in the genome sequence we excluded those sequences which also contained a run of 8 or more A nucleotides in the genome at the position of the putative splice site (n = 1968). 100 bp of coding sequence at each end was also included so that the start and stop codons were included in the analysis.

### A tiling approach to analyse larger sequences

Using the Bayesian approach outlined above, the amount of time taken to analyse a given sequence increases exponentially with sequence length, making analysis of larger sequences or chromosomes as a single DNA sequence difficult. However, since our method is sensitive to changes in composition occurring over relatively short stretches of DNA it is not necessary to analyse larger sequences in one pass. Therefore as computational demand is exponentially related sequence length but linearly related to the number of sequences of the same length we chose to adopt a tiling approach for analysing large sequences. We used this tiling approach to segment the large multi-copy gene arrays. In this tiling approach, the multi-copy array was split up into overlapping sequences each of 500 nucleotides. A 500 bp sequence tile was taken every 20 nucleotide positions so that each tile had a 460 bp overlap with either neighbour sequence tile. This means that each position in the large sequence occurred in 25 overlapping sequence tiles. Each sequence tile was subjected to Bayesian segmentation analysis and the probability profile for the query sequence was re-constructed from the mean probability of all overlapping profiles.

## Results

### A Bayesian segmentation method detects simulated changes in nucleotide composition

**Figure 1:**
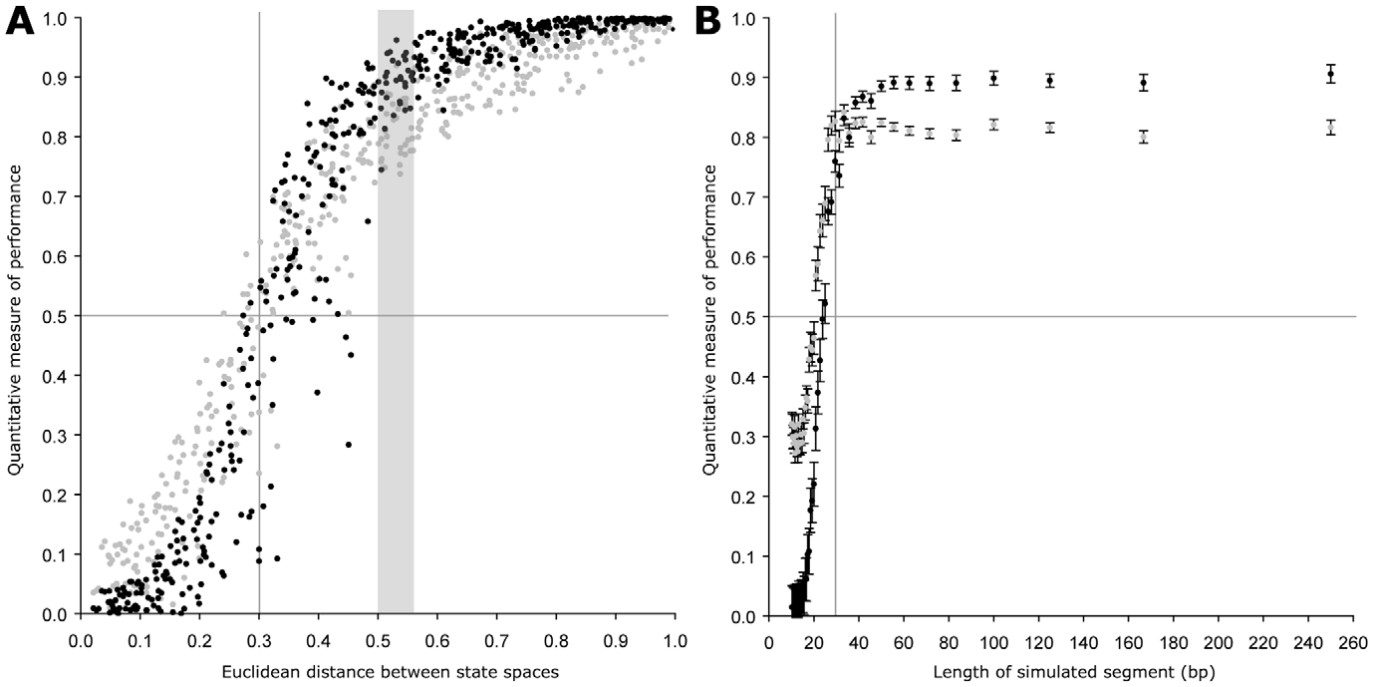
Testing the performance of the Bayesian segmentation method to changes in nucleotide composition and sequence length. **A**) The effect of state space difference on precision and recall of the Bayesian segmentation method to identify multiple change points in sequence composition for a fixed segment length. Grey dots represent the precision score black dots represent the recall score. The shaded area (0.50–0.55) indicates the interval chosen for further analysis in part B. **B**) The effect of segment length on the precision and recall performance of the method to identify change points in sequence composition for a set interval of state space difference. Grey dots represent the mean precision score, and black dots represent the mean recall score for 100 replicates made at each segment length. Error bars indicate 1 standard error of the mean.

Several tests were designed to determine the sensitivity of the Bayesian segmentation method to changes in sequence composition. The first test entailed simulation of 100 DNA sequences each composed of 10 directly abutted segments of 50 bp. Each segment was simulated using one of two randomly generated state spaces so that each segment (excluding those at the ends) was positioned between two segments of contrasting composition. The Euclidean distance between the two contrasting state spaces was recorded. For example if the state space for segment 1, 3, 5, 7 and 9 was A = 0.25, C = 0.25, G = 0.25, T = 0.25 and the state space for segments 2, 4, 6, 8 and 10 was A = 0.30, C = 0.20, G = 0.21, T = 0.29 then the Euclidean distance between these state spaces is 0.09. Each of the 100 simulated sequences was then subject to Bayesian segmentation. The most likely number of change points (N) given the sequence was inferred and the corresponding N most likely change points were selected (Figure 1A). The positions of the inferred change points were noted in each of the 100 simulated sequences and the number of change points within 5 bp of the true change point between segments was recorded. This process was repeated 500 times for different randomly generated state spaces so that the relationship between sequence difference and detection performance could be determined (Figure 1A). This analysis reveals that for a change in sequence composition of ≥0.5 the method achieves both high precision and recall (≥0.82 and ≥0.88 respectively). i.e. there is a false positive detection rate of ≤0.18 and a false negative detection rate of ≤0.12.

To assess how the performance was affected by the length of the simulated sequence segments, we simulated 10 sequences each subdivided into several segments of alternating nucleotide composition. As before, the state space for each segment was randomly generated. However, the Euclidean distance between segments was constrained to lie on the interval [0.50, 0.55] where the mean precision and recall were 0.82 and 0.88 respectively (Figure 1A). As above, each segment (excluding those at the ends) is positioned between segments of contrasting composition. The results for 100 replicates for each segment size are shown in Figure 1B. This showed that there is a substantial decline in both the precision and recall scores for the segmentation method for simulated segments below 30 bp in length. Moreover, for simulated sequences of 20 bp or fewer, <50% of the simulated changes in composition are correctly identified.

### Analysing larger sequences: Comparative analysis of monotypic and polytypic gene arrays

**Figure 2:**
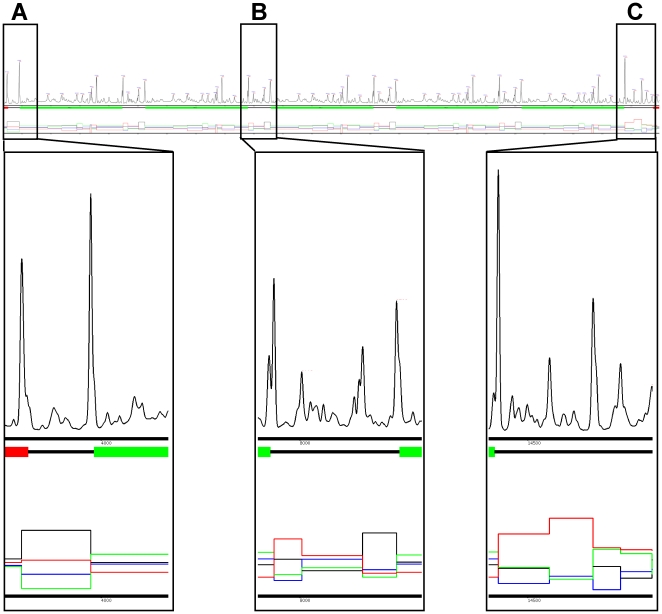
The Trypanosoma brucei PFR1 gene array on chromosome 3. Directly underneath the profile a box diagram of the genes is indicted with the PFR1 genes coloured in green and the up- and downstream genes coloured in red, intergenic regions are indicated by a black line. Underneath this the base composition of the segments is shown as a line chart where the red line represents the frequency of T residues, the black line represents the frequency of A residues, the blue line represents the frequency of C residues and the green line represents the frequency of G residues observed in the region between peaks. **A**) The 5′ end of the gene array. **B**) The region between two PFR1 coding sequences. **C**) The 3′ end of the gene array.

To test the approach on real sequence data we applied the method to several well characterised monotypic and polytypic gene arrays from the *Trypanosoma brucei* genome for which some regulatory information is already known (PFR1, PFR2, Histone H2A, H2B, H3, H4 and the α-β tubulin array). This information includes some of the life-cycle stages in which these genes are expressed and the positions of some experimentally characterised regulatory sites. The PFR1 gene array is used as an illustrative example in Figure 2. However, full gene arrays can be found in file S3, S4, S5 & S6 for PFR1, PFR2, Alpha-Beta tubulin and histone H4 respectively. All 7 interrogated gene arrays were found to be preceded by a large A-rich segment of DNA (Figure 2A, file S3, S3, S5, S6) and are followed by one or more large T-rich segments (Figure 2C, file S3, S4, S5, S6). The one exception to this rule is the tubulin gene array (file S5), as this array is not followed by any predominantly T-rich segments. However the tubulin array is directly followed by the histone H3 array which ends with several large T-rich segments. If these two adjacent arrays are considered to be a single large array, then they fit the pattern exhibited by the other arrays i.e. being directly preceded by at least one large A-rich segment and followed by at least one large T-rich segment.

### Three segment types occur in every arrayed gene intergenic region

The region between the coding sequences of each array is predicted to be composed of several distinct segments of DNA. We found that, mirroring the structure of the array itself, each coding sequence within the array is preceded by an A-rich segment and followed by a T-rich segment. However, between these two oppositely composed segments the number and type of segment found varies. In the case of the PFR1 intergenic region, there is only one additional segment which is pyrimidine rich, i.e. composed predominantly of T and C residues (Figure 2B). In contrast to this, the PFR2 (file S4), alpha-to-beta (file S5), beta-to-alpha (file S5) and histone H4 (file S6) intergenic regions contain 3, 3, 7 and 4 segments respectively. Comparison of the segments identified by this analysis revealed that as well as the two common segments described above, one additional segment type composed predominantly of T and C residues is also common to all of the arrays. In each case, this additional common segment directly precedes the A-rich segment in each intergenic region (Figure 2B, file S3, S4, S5, S6).

### Splice-acceptor sites are found at the boundary between the A-rich and pyrimidine-rich segments

Interrogation of the sequence present at the predicted change point between the A-rich segment and the preceding TC-rich segment in each array revealed the presence of an AG di-nucleotide. From experimental data, each of the identified AG di-nucleotides is known to be the trans-splice site used for each of the analysed genes. While there is a strong signal detectable by Bayesian segmentation which is associated with trans-splice sites, there does not appear to be any correlation between polyadenylation sites or branch-point sites and predicted segment boundaries. For example, the polyadenylation site experimentally characterised for the PFR1 gene [51] does not correspond to any peak and but rather occurs within the pyrimidine-rich segment, 44 bp downstream of the T-rich segment 3′ boundary. Similarly for PFR2 [51] the polyadenylation site does not correspond with any predicted change point but is 106 bases downstream of the 3′ boundary of the T-rich segment following the open reading frame and occurs before the common pyrimidine-rich segment.

### Interrogation of interaction potential

The occurrence of segments of similar size and opposing composition at opposite ends of the arrayed gene mRNAs is intriguing, and raises the question as to whether these segments of RNA could interact following transcription. This is interesting as secondary structure can act to either enhance or reduce the stability and hence abundance of an RNA molecule within the cell. The potential hybridisation of these two segments was interrogated *in silico* using the DINAMelt Server [52] for both the PFR1 and PFR2 genes (File S7). It is unknown whether an interaction between these oppositely composed segments occurs *in vivo*. However it is interesting to note that, for both PFR1 and PFR2, there is potential for these oppositely composed segments surrounding the coding sequence to hybridise with extensive base pairing (file S7). If these interactions were to occur, they would be highly stable at temperatures relevant to both life-cycle stages of the parasite (file S7). While it is interesting to consider the interaction potential of these gene encompassing DNA sequences, there is no genome-wide correlation between transcript level as determined by RNAseq technology [50] and the free energy of interaction of the DNA immediately surrounding each gene (file S8). As interaction between the ends of a single RNA molecule might provide a function independent of modulating transcript abundance, we interrogated whether free energy of hybridisation was higher for sequences encompassing the same gene than for sequences from the ends of different randomly selected genes. This analysis showed that there is no difference between the distribution of free energies observed for hybridisation of the 3′ and 5′ ends of the same molecule and the distribution of free energies observed from randomly selected 3′ and 5′ ends from different molecules (file S8). Additionally, the distribution of free energies observed for hybridisation of the 3′ and 5′ ends is more positive (and hence less stable) for randomly generated sequences (file S8). This analysis suggests that the ends of the RNA molecule are coded in such a way as to minimise their interaction potential. Hence, it is unlikely that there is a role played by hybridisation between the 3′ and 5′ un-translated regions in regulating mRNA level control or gene expression regulation in general.

### Genome-wide analysis of regulatory element detection

To determine whether our observations from the monotypic and polytypic gene arrays described above apply to the whole genome we utilised a recently produced high-throughput mRNA sequence dataset [50]. From the complete set of intergenic sequences predicted in the *T. brucei* genome we extracted all nucleotide sequences spanning intergenic regions in the direction of transcription (n = 10,051). We filtered this set to so that only intergenic sequences which had both annotated tans-splice sites (n = 6,577) and polyadenylation sites (n = 5,650) were retained. This filtration resulted in the selection of 5,121 intergenic regions. We included 100 bp of coding sequence at each end such that our predicted intergenic regions contained start and stop codons ad defined positions. We subjected these intergenic regions to Bayesian segmentation. The most probable number of change points given the sequence was determined and the corresponding most probable change points selected. We then counted the number of times that each of the characterised features in the intergenic region (comprising the start codon, the stop codon, the polyadenylation site and the splice acceptor site) was within 5 bp of an inferred change point in nucleotide composition (Table 1). We found that approximately one third of all splice sites analysed occur within 5 bp of a predicted change in nucleotide composition. In contrast to this only 17% of polyadenylation sites occur within 5 bp of a change in nucleotide composition, which is similar to background levels (16.4%). The change in composition associated with the trans-splice site is more readily detectable by this sequence segmentation method than the change in composition between coding and non-coding sequence as both the start and stop codons are only detected by this method in 23.2% and 20.8% of intergenic regions respectively (Table 1).

**Table 1:** Genome wide analysis of intergenic region features.

Nucleotide element	Proportion of sites at change-points
Trans splice site	0.334
Polyadenylation site	0.17
Start codon	0.232
Stop codon	0.208
Randomly selected position	0.164

The score given represents the proportion of 5,121 elements which were identified as inferred change points in nucleotide composition.

### Genomic environment of polyadenylation sites

**Figure 3:**
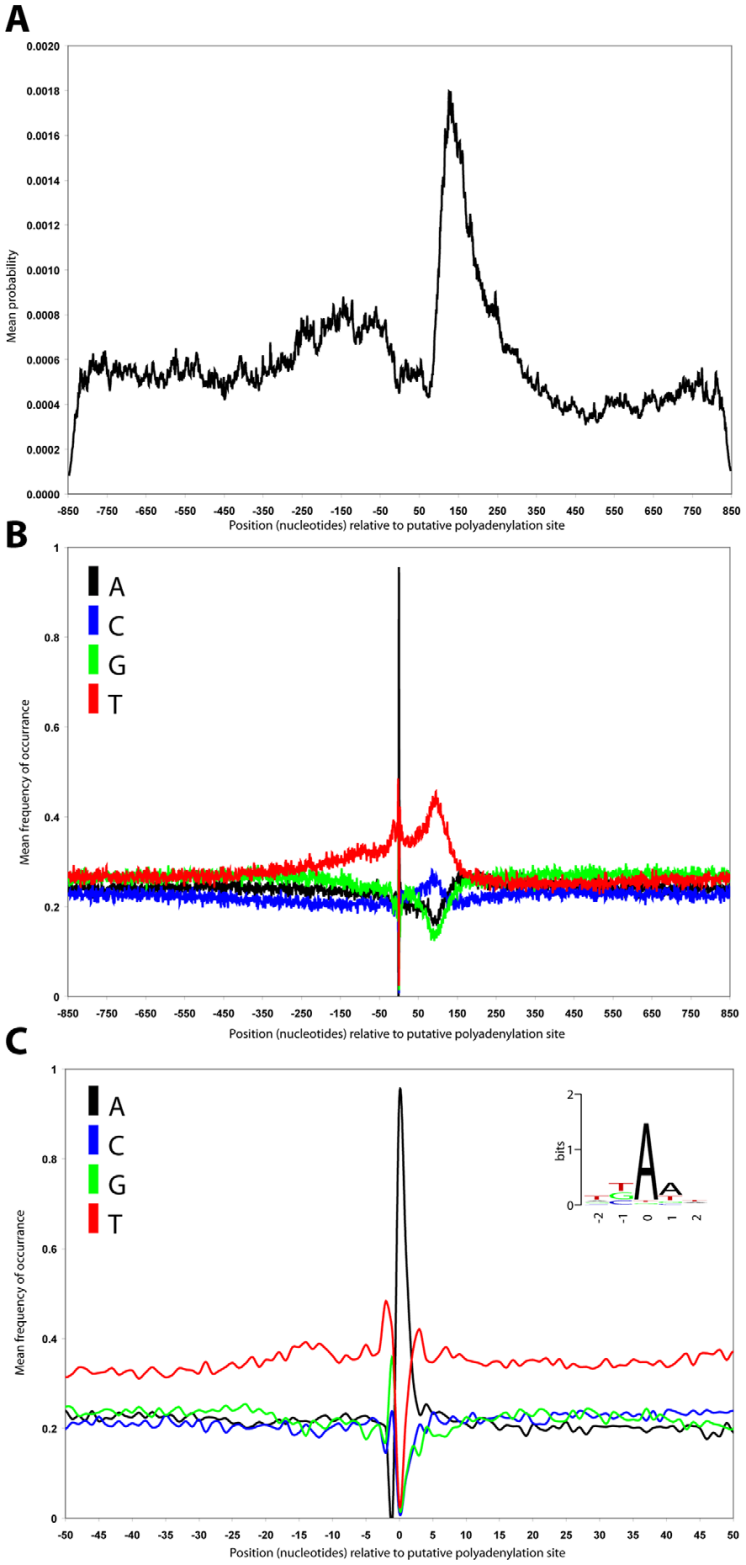
A genome wide analysis of polyadenylation sites. **A**) Averaged probability density profiles for 4037 putative polyadenylation sites centred on the putative polyadenylation site. **B**) Averaged sequence composition of 1700 nucleotides surrounding the putative polyadenylation site for the 4037 putative polyadenylation sites. **C**) Average sequence composition of the 40 nucleotides immediately surrounding the putative polyadenylation site. The black, blue, green and red lines represent the frequencies of A, C, G and T residues respectively.

To confirm the above observations that polyadenylation sites do not seem to occur at predicted changes in nucleotide composition a further analysis was performed. For each of the polyadenylation sites, 1,700 bp centred on the putative polyadenylation site was extracted from genome sequence in the same orientation as the direction of the coding sequence. Each of the sequences was then analysed by Bayesian segmentation and the resulting probability distributions averaged (Figure 3A). This analysis shows that, in agreement with our analysis of arrayed genes and subsequent genome wide analysis, there is no detectable change in nucleotide composition that is associated with the position of polyadenylation sites (Figure 3A).

However, at the location of the polyadenylation site, there is a 4 nucleotide motif “the TKAA motif” (where K = T or G) (Figure 3C). This short degenerate motif bears some similarity to the consensus polyadenylation motif utilised by fungi, metazoa and land plants - AATAAA. It appears that it is, on average, located in a local maximum of T-nucleotide frequency which is also a local minimum in both A and G residue frequency. Downstream of the polyadenylation site there is a pronounced decrease in the observed frequency of A and G nucleotides which is offset by a sharp rise in the frequency of observation of T nucleotides and a less pronounced increase in the frequency of C nucleotides. C nucleotides briefly continue to be present at higher frequencies before returning to a lower level at position +140. This level is slightly elevated relative to the region prior to the polyadenylation site.

At position +140 (+/− 12 bp) relative to the polyadenylation site there is an inversion in sequence composition as the frequencies of A and G nucleotides increase and the frequencies T and C nucleotides dramatically drop. This inversion correlates with a strong predicted change in sequence composition detected by Bayesian segmentation at the same position (Figure 3A). This composition inversion is strongly suggestive of a secondary-structure, and mirrors the composition inversion we identified in the arrayed genes at the position of the splice acceptor site.

### Genomic environment of trans-splice sites

**Figure 4:**
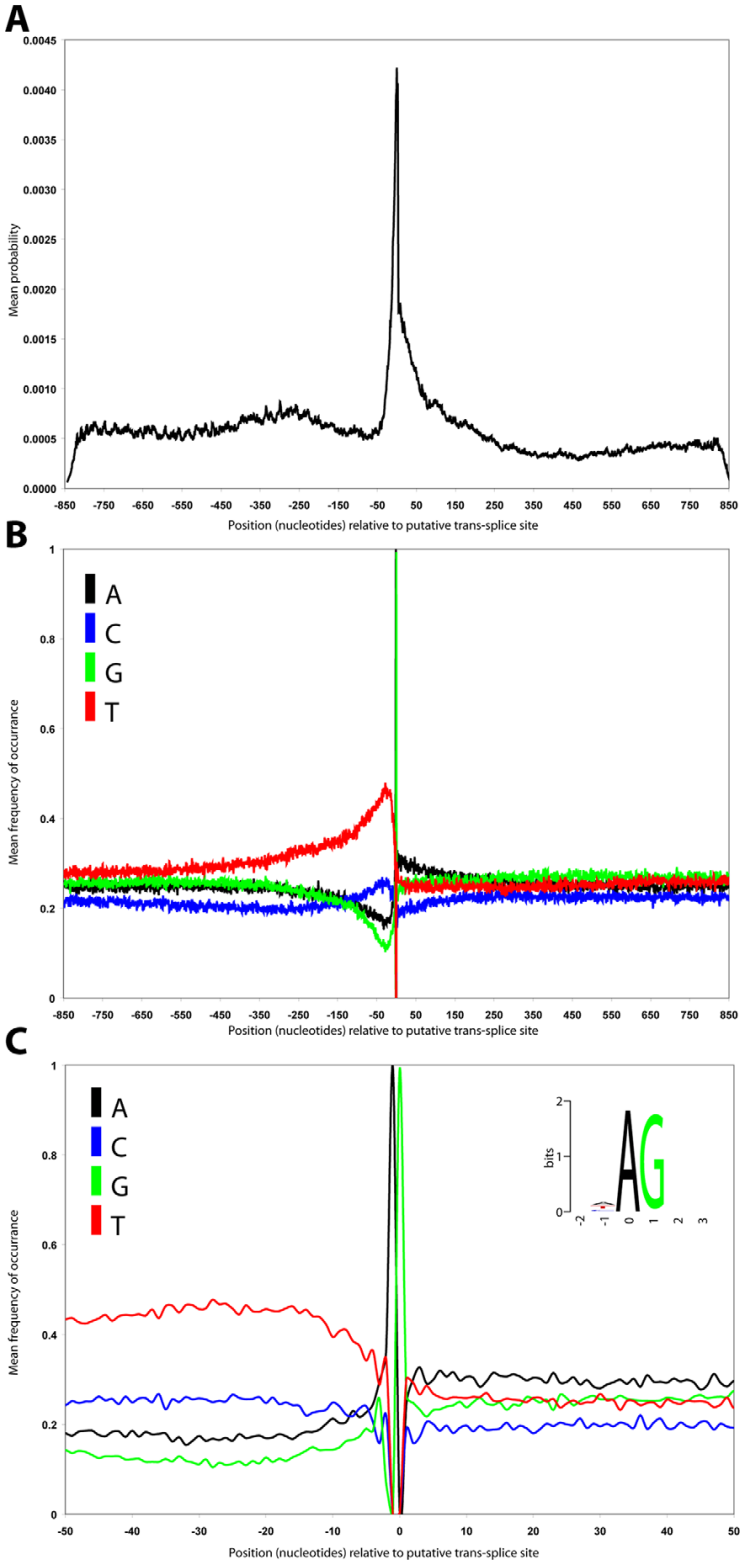
A genome wide analysis of trans-splice sites. **A**) Averaged probability density profiles for 5201 putative trans-splice sites centred on the putative trans-splice site. **B**) Averaged sequence composition of 1700 nucleotides surrounding putative trans-splice site for the 5201 putative sites. **C**) Average sequence composition of the 40 nucleotides immediately surrounding the putative trans-splice site. The black, blue, green and red lines represent the frequencies of A, C, G and T residues respectively.

To discover if the composition inversion that we observed for the arrayed genes at the splice acceptor site (which occurs on average at position +140 relative to the polyadenylation site in all genes) is associated with trans-splice sites on a genome wide scale we again turned to the high-throughput sequence data [50]. Trans-splice sites were selected and interrogated in the following manner. As for the polyadenylation sites, 1,700 bp centred on the putative trans-splice site was extracted from genome sequence in the same orientation of the direction of the transcript. Each of the selected sequences was then analysed by Bayesian segmentation and the resulting probability distributions averaged (Figure 4). This analysis shows that, as we had seen in our analysis of the arrayed genes and our genome wide analysis of intergenic regions there is on average a change in sequence composition detectable by Bayesian segmentation which occurs at the position of the putative trans-splice site (Figure 4A).

Unlike the polyadenylation sites, at the location of this change point in sequence composition there is no detectable recurring motif in addition to the AG acceptor di-nucleotide (Figure 4C). Throughout the region upstream of the putative trans-splice site the sequence is predominantly T-rich. As you approach the trans-splice site from the upstream region the sequence becomes increasingly T-rich and G-poor with a local maxima/minima occurring at approximately position −30. As observed in our analysis of arrayed genes the splice acceptor site occurs at an inversion in sequence composition where A and G nucleotides replace T and C nucleotides as the highest frequency representatives. This composition inversion strongly suggests that secondary structure, rather than specific sequence elements, are driving trans-splice site delineation.

## Discussion

The identification of regulatory elements within DNA, RNA and protein sequences is key to progressing our understanding of the developmental, morphological and behavioural diversity of life. The trypanosomatid transcription system has provided a wealth of insight into transcription processes in eukaryotes yet its regulation remains largely uncharacterised. Evidence from analyses of protein components which mediate transcription suggest that the system of regulation in *Trypanosoma brucei* is likely to be dramatically different to well documented systems such as in humans and yeast. Hence, there is a need to develop *ab initio* methods for regulatory information discovery in this organism.

In our analysis, we found several features common to the genes which are located in multi-copy gene arrays. First, each array is directly preceded by a large A-rich segment of DNA. Each array is also directly followed by at least one large T-rich segment of DNA. Interestingly, the tubulin gene array appears to be the exception to this rule as it is not followed by a T-rich segment. However, it is followed by an additional array containing the histone H3 genes which itself is immediately followed by several large segments of T-rich DNA. The opposing composition found at the opposite ends of the arrays is also found surrounding the individual genes of the arrays themselves. In this case, each coding unit is preceded by an A-rich segment and followed by a T-rich segment. Interestingly the 5′ boundary of the A-rich segment corresponds to an AG di-nucleotide (+/− 3 bp) which in the case of all analysed arrayed genes is known to be the AG used *in vivo*. In each case, this predicted 5′ boundary separates the A-rich segment from a preceding pyrimidine rich segment. The fact that this critical regulatory site occurs at sequence boundaries raises the question as to what functions the other sequence boundaries might be playing in these intergenic regions.

T-rich sequences are known to cause pausing which can lead to transcriptional termination by RNA polymerase III in humans and in yeast [53]. Indeed the T-rich tracts in the spliced leader RNA genes have been shown to be sufficient to terminate transcription by Pol II in trypanosomatids [54]. Therefore, one possibility is that these T-rich regions at the ends of arrays may function as transcriptional modulators. Hence we propose these T-rich regions may be causing transcriptional pausing to aid post-transcriptional processing of transcribed genes. It is possible that pausing leading to termination may be dependent on the length of the T-rich tract.

The opposite composition of the 5′ and 3′ ends of the mRNA molecule raises several interesting possibilities for a potential role in gene expression regulation. One obvious possibility for the role of oppositely composed segments encompassing each coding sequence is in producing secondary structure in the mRNA. As there is no correlation between the observed mRNA level in the cell and the free energy of interaction between these opposing ends, we can infer that this putative secondary structure is not influencing transcript abundance in the cell. However a role in expression regulation independent of transcript abundance cannot be ruled out.

By providing a genome wide analysis of intergenic regions we showed that approximately 1/3 of all trans-splice sites occur within 5 bp of an inferred change in nucleotide composition. Moreover, by interrogating the DNA surrounding putative polyadenylation and trans-splice sites we have demonstrated that our observations for the highly expressed arrayed genes extends to all sampled genes in *T. brucei*. We also identified a common polyadenylation motif “TKAA.” Unlike in metazoa, fungi and land plants, the *T. brucei* polyadenylation motif is short and degenerate. We propose that the degeneracy of this motif may be due to a change in the role of the polyadenylation motif from a cleavage site delineator to a coding sequence protector. As the free 3′ end of an RNA molecule is generated by trans-splicing of the downstream gene there is no requirement for the polyadenylation machinery to produce its own 3′ end. In the presence of a near by free 3′ end, the function of the polyadenylation machinery is then to protect the coding sequence from destruction by exonucleases through addition of the poly(A) tail. This modified role of the polyadenylation signal is hence different in trypanosomatids than in other eukaryotes. In this context, it is interesting to note that the polyadenylation motif we identify encompasses two of the three stop codons (TGA and TAA) and that these two stop codons are the two most commonly used stop codons in the *T. brucei* genome (TGA = 37%, TAA = 35% and TAG = 28%).

There is an inversion in nucleotide composition which occurs at the position of the trans-splice site. We propose that this inversion in composition is the biological mechanism of trans-splice site specification which is being utilised by the cell. Moreover, this composition inversion suggests a mechanism which is dependent on the secondary structure of the emerging RNA molecule around this inversion point. This proposal is supported by the observations that there are no recurring motifs or specific/degenerate sequence elements associated with the trans-splice site.

## Supporting Information

File S1 **The Bayesian segmentation method.**
(PDF)

File S2 **The source code for the Perl algorithm which subjects FASTA format nucleotide sequences to Bayesian segmentation.**
(PL) Click here for additional data file.

File S3 **The Trypanosoma brucei PFR1 gene array on chromosome 3.** In each case the peak base and the three bases either side are written in blue and centred above the peak. The peak position from the start of the sequence is given in red. Directly underneath the profile a box diagram of the genes is indicated with the beta tubulin genes coloured in green and the alpha tubulin genes coloured in blue. The up- and downstream genes are coloured in red, intergenic regions are indicated by a black line. Underneath this the base composition of the segments is shown as a line chart where the red line represents the frequency of T residues, the black line represents the frequency of A residues, the blue line represents the frequency of C residues and the green line represents the frequency of G residues observed in the region between peaks.
(TIF)

File S4 **The Trypanosoma brucei PFR2 gene array on chromosome 8.** In each case the peak base and the three bases either side are written in blue and centred above the peak. The peak position from the start of the sequence is given in red. Directly underneath the profile a box diagram of the genes is indicated with the beta tubulin genes coloured in green and the alpha tubulin genes coloured in blue. The up- and downstream genes are coloured in red, intergenic regions are indicated by a black line. Underneath this the base composition of the segments is shown as a line chart where the red line represents the frequency of T residues, the black line represents the frequency of A residues, the blue line represents the frequency of C residues and the green line represents the frequency of G residues observed in the region between peaks.
(TIF)

File S5 **The Trypanosoma brucei tubulin gene array on chromosome 3.** In each case the peak base and the three bases either side are written in blue and centred above the peak. The peak position from the start of the sequence is given in red. Directly underneath the profile a box diagram of the genes is indicated with the beta tubulin genes coloured in green and the alpha tubulin genes coloured in blue. The up- and downstream genes are coloured in red, intergenic regions are indicated by a black line. Underneath this the base composition of the segments is shown as a line chart where the red line represents the frequency of T residues, the black line represents the frequency of A residues, the blue line represents the frequency of C residues and the green line represents the frequency of G residues observed in the region between peaks.
(TIF)

File S6 **The Trypanosoma brucei histone H4 gene array on chromosome 5.** In each case the peak base and the three bases either side are written in blue and centred above the peak. The peak position from the start of the sequence is given in red. Directly underneath the profile a box diagram of the genes is indicated with the histone H4 genes coloured in green. The intergenic regions are indicated by a black line. Underneath this the base composition of the segments is shown as a line chart where the red line represents the frequency of T residues, the black line represents the frequency of A residues, the blue line represents the frequency of C residues and the green line represents the frequency of G residues observed in the region between peaks.
(TIF)

File S7 **The predicted hybridisation structure for the A-rich and T(U)-rich segments surrounding the PFR1 and PFR2 open reading frames.** Structures predicted using the DINAmelt server.
(TIF)

File S8 **The free energy of hybridisation between the DNA immediately encompassing the coding sequence.** A) Correlation between transcript level in bloodstream form parasites and free energy of hybridisation at 37°C. B) Correlation between transcript level in procyclic form parasites and free energy of hybridisation at 28°C. C) Comparison of distribution of free energies between paired ends (white circles) and random ends (black boxes) and randomly generated sequence (red circles).
(PDF)

## References

[1] Zheng ZM (2004). Regulation of alternative RNA splicing by exon definition and exon sequences in viral and mammalian gene expression.. J Biomed Sci.

[2] Wang ZF, Whitfield ML, Ingledue TC, Dominski Z, Marzluff WF (1996). The protein that binds the 3′ end of histone mRNA: a novel RNA-binding protein required for histone pre-mRNA processing.. Genes Dev.

[3] Gustafsson C, Govindarajan S, Minshull J (2004). Codon bias and heterologous protein expression.. Trends Biotechnol.

[4] Lavner Y, Kotlar D (2005). Codon bias as a factor in regulating expression via translation rate in the human genome.. Gene.

[5] Hamilton PB, Gibson WC, Stevens JR (2007). Patterns of co-evolution between trypanosomes and their hosts deduced from ribosomal RNA and protein-coding gene phylogenies.. Mol Phylogenet Evol.

[6] Simpson AG, Gill EE, Callahan HA, Litaker RW, Roger AJ (2004). Early evolution within kinetoplastids (euglenozoa), and the late emergence of trypanosomatids.. Protist.

[7] Hoare CA (1929). Studies on Trypanosoma grayi.. Transactions of the Royal Society of Tropical Medicine and Hygiene.

[8] Camargo EP (1999). Phytomonas and other trypanosomatid parasites of plants and fruit.. Advances in Parasitology.

[9] Benne R, Van den Burg J, Brakenhoff JP, Sloof P, Van Boom JH (1986). Major transcript of the frameshifted coxII gene from trypanosome mitochondria contains four nucleotides that are not encoded in the DNA.. Cell.

[10] Boothroyd JC (1985). Antigenic variation in African trypanosomes.. Annu Rev Microbiol.

[11] Borst P (1986). Discontinuous transcription and antigenic variation in trypanosomes.. Annu Rev Biochem.

[12] Van der Ploeg LH (1986). Discontinuous transcription and splicing in trypanosomes.. Cell.

[13] Rudenko G, Bishop D, Gottesdiener K, Van der Ploeg LH (1989). Alpha-amanitin resistant transcription of protein coding genes in insect and bloodstream form Trypanosoma brucei.. EMBO J.

[14] Navarro M, Gull K (2001). A pol I transcriptional body associated with VSG mono-allelic expression in Trypanosoma brucei.. Nature.

[15] Brown SD, Huang J, Van der Ploeg LH (1992). The promoter for the procyclic acidic repetitive protein (PARP) genes of Trypanosoma brucei shares features with RNA polymerase I promoters.. Mol Cell Biol.

[16] Janz L, Clayton C (1994). The PARP and rRNA promoters of Trypanosoma brucei are composed of dissimilar sequence elements that are functionally interchangeable.. Mol Cell Biol.

[17] Pham VP, Qi CC, Gottesdiener KM (1996). A detailed mutational analysis of the VSG gene expression site promoter.. Mol Biochem Parasitol.

[18] Sherman DR, Janz L, Hug M, Clayton C (1991). Anatomy of the parp gene promoter of Trypanosoma brucei.. Embo J.

[19] Vanhamme L, Pays A, Tebabi P, Alexandre S, Pays E (1995). Specific binding of proteins to the noncoding strand of a crucial element of the variant surface glycoprotein, procyclin, and ribosomal promoters of trypanosoma brucei.. Mol Cell Biol.

[20] Gilinger G, Bellofatto V (2001). Trypanosome spliced leader RNA genes contain the first identified RNA polymerase II gene promoter in these organisms.. Nucleic Acids Res.

[21] Engstler M, Boshart M (2004). Cold shock and regulation of surface protein trafficking convey sensitization to inducers of stage differentiation in Trypanosoma brucei.. Genes Dev.

[22] Estevez AM (2008). The RNA-binding protein TbDRBD3 regulates the stability of a specific subset of mRNAs in trypanosomes.. Nucleic Acids Res.

[23] Walrad P, Paterou A, Acosta-Serrano A, Matthews KR (2009). Differential trypanosome surface coat regulation by a CCCH protein that co-associates with procyclin mRNA cis-elements.. PLoS Pathog.

[24] Furger A, Schurch N, Kurath U, Roditi I (1997). Elements in the 3′ untranslated region of procyclin mRNA regulate expression in insect forms of Trypanosoma brucei by modulating RNA stability and translation.. Mol Cell Biol.

[25] Schurch N, Furger A, Kurath U, Roditi I (1997). Contributions of the procyclin 3′ untranslated region and coding region to the regulation of expression in bloodstream forms of Trypanosoma brucei.. Mol Biochem Parasitol.

[26] McDonagh PD, Myler PJ, Stuart K (2000). The unusual gene organization of Leishmania major chromosome 1 may reflect novel transcription processes.. Nucleic Acids Res.

[27] Martinez-Calvillo S, Yan S, Nguyen D, Fox M, Stuart K (2003). Transcription of Leishmania major Friedlin chromosome 1 initiates in both directions within a single region.. Mol Cell.

[28] Wright JR, Siegel TN, Cross GA Histone H3 trimethylated at lysine 4 is enriched at probable transcription start sites in Trypanosoma brucei.. Mol Biochem Parasitol.

[29] Siegel TN, Hekstra DR, Kemp LE, Figueiredo LM, Lowell JE (2009). Four histone variants mark the boundaries of polycistronic transcription units in Trypanosoma brucei.. Genes Dev.

[30] Ivens AC, Peacock CS, Worthey EA, Murphy L, Aggarwal G (2005). The genome of the kinetoplastid parasite, Leishmania major.. Science.

[31] Clayton CE (2002). Life without transcriptional control? From fly to man and back again.. Embo J.

[32] Berriman M, Ghedin E, Hertz-Fowler C, Blandin G, Renauld H (2005). The genome of the African trypanosome Trypanosoma brucei.. Science.

[33] El-Sayed NM, Myler PJ, Bartholomeu DC, Nilsson D, Aggarwal G (2005). The genome sequence of Trypanosoma cruzi, etiologic agent of Chagas disease.. Science.

[34] Vanhamme L, Pays E (1995). Control of gene expression in trypanosomes.. Microbiol Rev.

[35] Ullu E, Matthews KR, Tschudi C (1993). Temporal order of RNA-processing reactions in trypanosomes: rapid trans splicing precedes polyadenylation of newly synthesized tubulin transcripts.. Mol Cell Biol.

[36] Hotz HR, Hartmann C, Huober K, Hug M, Clayton C (1997). Mechanisms of developmental regulation in Trypanosoma brucei: a polypyrimidine tract in the 3′-untranslated region of a surface protein mRNA affects RNA abundance and translation.. Nucleic Acids Res.

[37] Hug M, Hotz HR, Hartmann C, Clayton C (1994). Hierarchies of RNA-processing signals in a trypanosome surface antigen mRNA precursor.. Mol Cell Biol.

[38] Lee MG, Van der Ploeg LH (1997). Transcription of protein-coding genes in trypanosomes by RNA polymerase I.. Annu Rev Microbiol.

[39] Siegel TN, Tan KS, Cross GA (2005). Systematic study of sequence motifs for RNA trans splicing in Trypanosoma brucei.. Mol Cell Biol.

[40] Gopal S, Awadalla S, Gaasterland T, Cross GA (2005). A computational investigation of kinetoplastid trans-splicing.. Genome Biol.

[41] Benz C, Nilsson D, Andersson B, Clayton C, Guilbride DL (2005). Messenger RNA processing sites in Trypanosoma brucei.. Mol Biochem Parasitol.

[42] Matthews KR, Tschudi C, Ullu E (1994). A common pyrimidine-rich motif governs trans-splicing and polyadenylation of tubulin polycistronic pre-mRNA in trypanosomes.. Genes Dev.

[43] LeBowitz JH, Smith HQ, Rusche L, Beverley SM (1993). Coupling of poly(A) site selection and trans-splicing in Leishmania.. Genes Dev.

[44] Cora D, Di Cunto F, Provero P, Silengo L, Caselle M (2004). Computational identification of transcription factor binding sites by functional analysis of sets of genes sharing overrepresented upstream motifs.. BMC Bioinformatics.

[45] Gupta M, Liu JS (2005). De novo cis-regulatory module elicitation for eukaryotic genomes.. Proc Natl Acad Sci U S A.

[46] Bailey TL, Boden M, Buske FA, Frith M, Grant CE (2009). MEME SUITE: tools for motif discovery and searching.. Nucleic Acids Res.

[47] Thompson W, Rouchka EC, Lawrence CE (2003). Gibbs Recursive Sampler: finding transcription factor binding sites.. Nucleic Acids Res.

[48] Liu JS, Lawrence CE (1999). Bayesian inference on biopolymer models.. Bioinformatics.

[49] Boys RJ, Henderson DA (2004). A Bayesian approach to DNA sequence segmentation.. Biometrics.

[50] Siegel TN, Hekstra DR, Wang X, Dewell S, Cross GA (2010). Genome-wide analysis of mRNA abundance in two life-cycle stages of Trypanosoma brucei and identification of splicing and polyadenylation sites.. Nucleic Acids Res.

[51] Deflorin J, Rudolf M, Seebeck T (1994). The major components of the paraflagellar rod of Trypanosoma brucei are two similar, but distinct proteins which are encoded by two different gene loci.. J Biol Chem.

[52] Markham NR, Zuker M (2005). DINAMelt web server for nucleic acid melting prediction.. Nucleic Acids Res.

[53] Schramm L, Hernandez N (2002). Recruitment of RNA polymerase III to its target promoters.. Genes Dev.

[54] Sturm NR, Yu MC, Campbell DA (1999). Transcription termination and 3′-End processing of the spliced leader RNA in kinetoplastids.. Mol Cell Biol.

