# Supplementary material for: Ab Initio Identification of Novel Regulatory Elements in the Genome of Trypanosoma brucei by Bayesian Inference on Sequence Segmentation
Source: PLoS One. 2011 Oct 3;6(10):e25666. doi: 10.1371/journal.pone.0025666 (PMC3185004; doi:10.1371/journal.pone.0025666)
Supplement: File S1 — The Bayesian segmentation method. (PDF) [file pone.0025666.s001.pdf]

Supplemental file 1

This program attempts to segment a given DNA sequence (R) into k distinct regions of homogeneous composition, where the segmentation (s), length (L) and composition (θ) of the homogeneous regions are unknown. The segmentation (s) is a conceptual which describes the order of segments within a sequence. Within each region $\theta = (\theta_A, \theta_C, \theta_G, \theta_T)$ and $\theta_A + \theta_C + \theta_G + \theta_T = 1$ so that θ_A is the unknown probability of observing an "A" residue at any given point in the region.

Segmentation proceeds by evaluating the probability that the margin between two nucleotides *(i & i+1)* is dividing segments of different composition, this is referred to hereafter as *P(i = change-point)*. To determine the probability *P(i = change-point)*, the marginal likelihood for the sequence (R) must first be computed. The marginal likelihood for any sequence, R, can be represented as the joint probability of $P(\kappa)$ (the probability that the sequence is composed of κ segments) multiplied by the sequence likelihood $P(R|\kappa)$ (the probability of obtaining sequence R from κ segments of composition θ^1, θ^2, θ^3, … θ^κ)

$$P(R) = \sum_{k=1}^{L} P(\kappa = k)\, P(R|\kappa = k) \qquad [1]$$

$P(\kappa)$ is simple to compute as *a priori* we consider all models with $\kappa \geq 1$ segments equally likely. Hence, $P(\kappa)$ is inversely proportional to κ_{max} (κ_{max} = L) for all values of κ. The number of segments (κ) cannot exceed the number of nucleotides in a sequence and must be greater than 0 (as a sequence cannot exist if is composed of 0 segments).

$$P(\kappa = k) = \frac{1}{L} \quad [2]$$

However, *P(R|κ)* is deceptively difficult to compute. The residues in each segment can be modelled by a variety of methods, but we chose to use a product multinomial model which describes the segmentation of the sequence R and a prior product Dirichlet model which describes its composition

$$P(R|\kappa = k) = P(s|\kappa = k)\, Dir\,(R|\kappa = k) \quad [3]$$

The product multinomial model in this instance is the number of segmentations of sequence R implied by segmentation S (*P(s|k)*). We consider all models of segmentation of a DNA sequence of length L with $\kappa \geq 1$ segments equally. Hence, as there are L – 1 possible change-points in a sequence of length L and there are κ - 1 possible change-points to allocate given κ segments, the prior probability for the segmentation of the sequence is inversely proportional to the number of ways to choose κ - 1 change-points from L – 1 possible change-points

$$P(s|\kappa = k) = \binom{L-1}{k-1}^{-1} \quad [4]$$

As DNA is generally considered to consist of four states, A, C, G and T, the simplest model for describing the composition of any given sequence would contain a single parameter which describes each state. The Dirichlet distribution parameters ($\alpha = (\alpha_A, \alpha_C, \alpha_G, \alpha_T)$) for each segment are independent of each other, and each segment is assumed to be independent of every other segment within the sequence. Hence, the prior product Dirichlet model for segment composition takes the form

$$Dir(R|\kappa = k) = \prod_k \frac{\Gamma(\sum_d \alpha_d)}{\prod_d \Gamma(\alpha_d)} \frac{\prod_d \Gamma(\theta_{k,d} L_k + \alpha_d)}{\Gamma(L_k + \sum_d \alpha_d)} \quad [5]$$

In this equation $\theta_{k,d}$ is the frequency of occurrence of residue type d in the kth segment, L_k is the length of the kth segment and α_d is the Dirichlet distribution parameter associated with residue type d. Hence, $P(R|\kappa = k)$ can be evaluated as

$$P(R|\kappa = k) = \sum_{|s|} \binom{L-1}{k-1}^{-1} \prod_k \frac{\Gamma(\sum_d \alpha_d)}{\prod_d \Gamma(\alpha_d)} \frac{\prod_d \Gamma(\theta_{k,d} L_k + \alpha_d)}{\Gamma(L_k + \sum_d \alpha_d)} \quad [6]$$

where $|s|$ is the number of possible segmentations implied by k. While evaluating $P(R|\kappa = 1)$ is simple using the above equation, it is difficult to compute $P(R|\kappa = k)$ for $k > 1$ directly. However a dynamic recursion approach can be employed (Auger & Lawrence 1989; Liu & Lawrence 1999). In this approach, the sequence R is considered to consist of two independent sub-sequences: $R1 = 1, ..., i$, and $R2 = i + 1, ..., L$. Hence $P(R|\kappa)$ can be described as the product of the two sub-sequences (for $0 < \delta < \kappa$)

$$P(R|\kappa) = P(R1|\kappa - \delta)\, P(R2|\delta) \quad [7]$$

As $P(R|\kappa = 1)$ can be evaluated directly from [6] for every subsequence of R, the dynamic recursion approach allows calculation of $P(R|\kappa)$ for increasing values of κ (for κ < L)

$$P(R|k) = \sum_{i=1}^{L} P(R_{[1:i]}|k-1)\, P(R_{[i+1:L]}|1) \quad [8]$$

Now, having calculated the probability that sequence R is obtained given it is composed of κ segments [8], the probability of there being κ segments [2], and the marginal

probability of the sequence R [1, 2 & 6] we can address the more interesting question of the probability that there are κ segments given the sequence R using Bayes' rule

$$P(\kappa|R) = \frac{P(R|\kappa)\,P(\kappa)}{P(R)} \qquad [9]$$

This equation is used in our analyses to determine the most likely number of change points contained in the sequence being analysed. Finally, using Bayes' rule again, the marginal probability that a change-point will occur at position *i* can be calculated. This is done by summing over all values of *κ*, the values obtained during dynamic recursion for every possible segmentation pattern either side of a boundary point between two nucleotides

$$P(\text{change–point} = i,\ \text{for } k|R) = \frac{1}{P(R)}\sum_{\kappa}\sum_{k} P(R_{[1:i]}|k)\,P(R_{[i+1:L]}|\kappa - k) \qquad [10]$$

The mathematics described above has been converted into a program called BaySeg which accepts files containing FASTA formatted sequences (the input files must not exceed 2GB). This program is written entirely in the Perl programming language for use with Perl 5.8.8 or later. It requires Perl to be compiled with both "threads" and "long-double" support enabled. It is written to run on UNIX/LINUX and may not run under other versions of Perl including Active Perl.

When the program terminates it outputs two files: The first, with the file extension .pkr, contains the posterior distribution of the number of change-points in the nucleotide sequence analysed (i.e. the marginal probabilities for the query sequence R for each possible κ the result obtained from [9]). In all analyses this file is used to determine the

most likely number of change points given the sequence. The second file, with the extension .pro, contains the marginal probability for each nucleotide being a change-point in sequence composition [10]. In all analyses this file was used to select the most probable change points. The program is supplied as Supplemental file 2.
