# Supplementary figures and images for: Ab Initio Identification of Novel Regulatory Elements in the Genome of Trypanosoma brucei by Bayesian Inference on Sequence Segmentation
Source: PLoS One. 2011 Oct 3;6(10):e25666. doi: 10.1371/journal.pone.0025666 (PMC3185004; doi:10.1371/journal.pone.0025666)

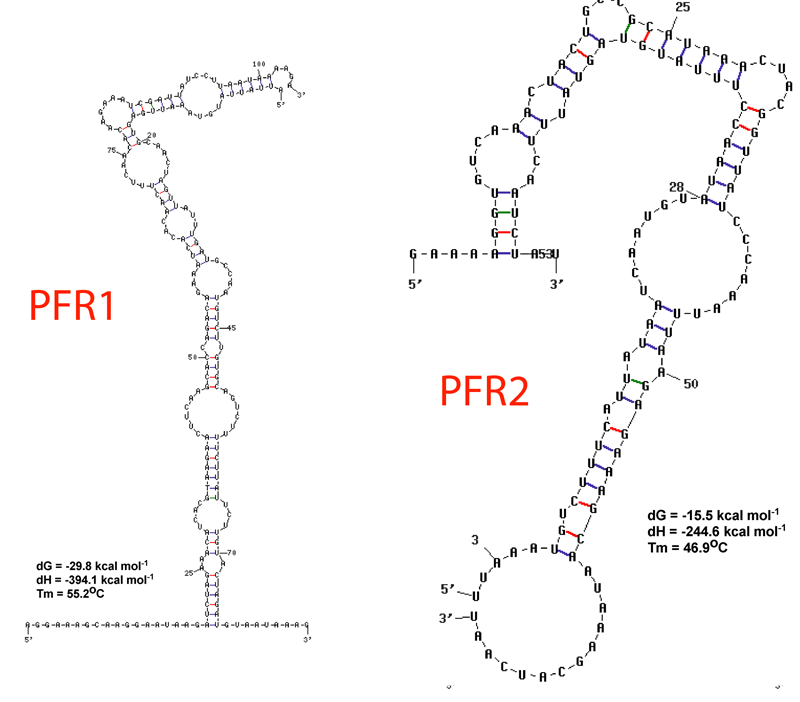

Supplement: File S7 — The predicted hybridisation structure for the A-rich and T(U)-rich segments surrounding the PFR1 and PFR2 open reading frames. Structures predicted using the DINAmelt server. (TIF) [file pone.0025666.s007.tif]

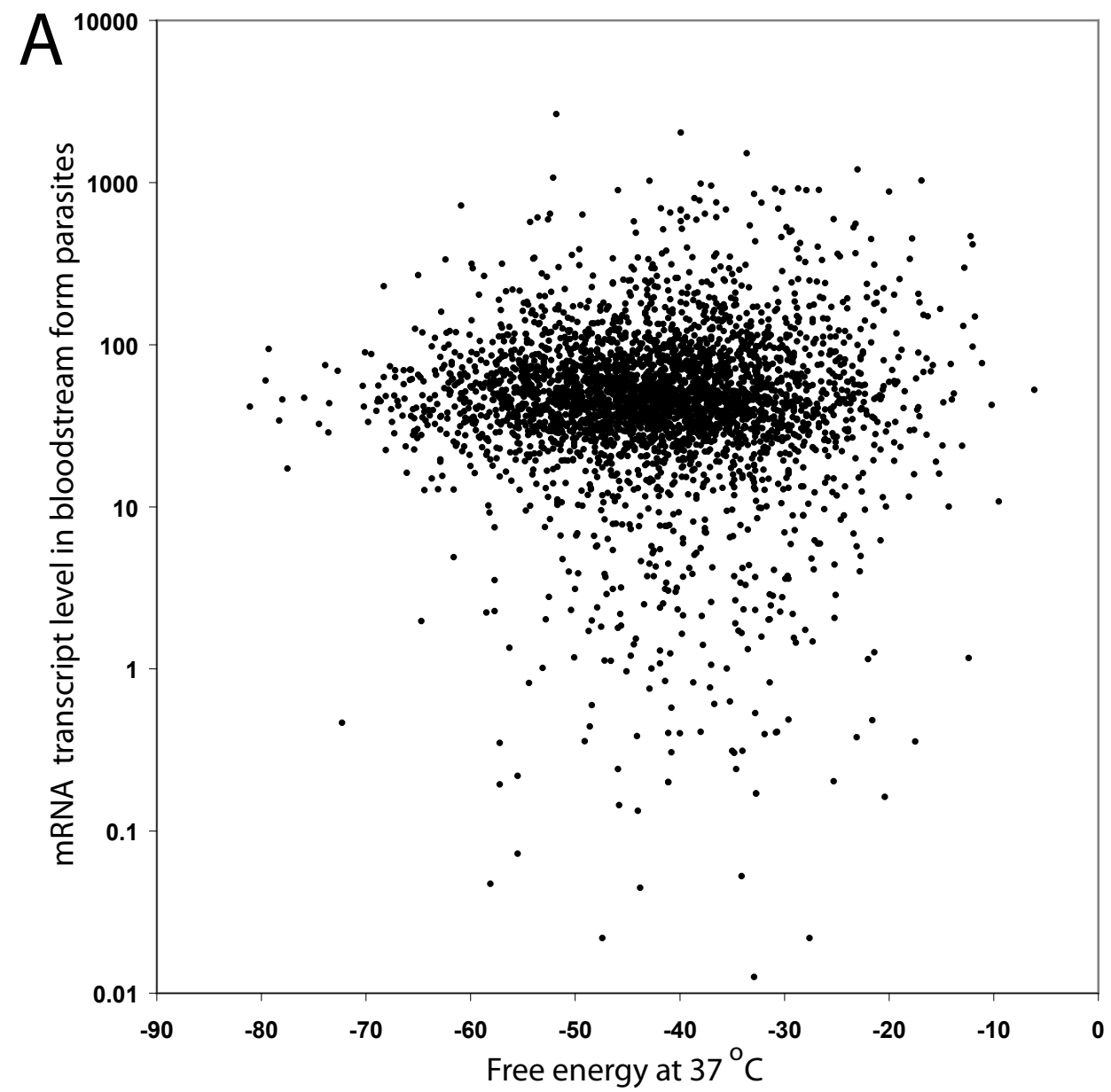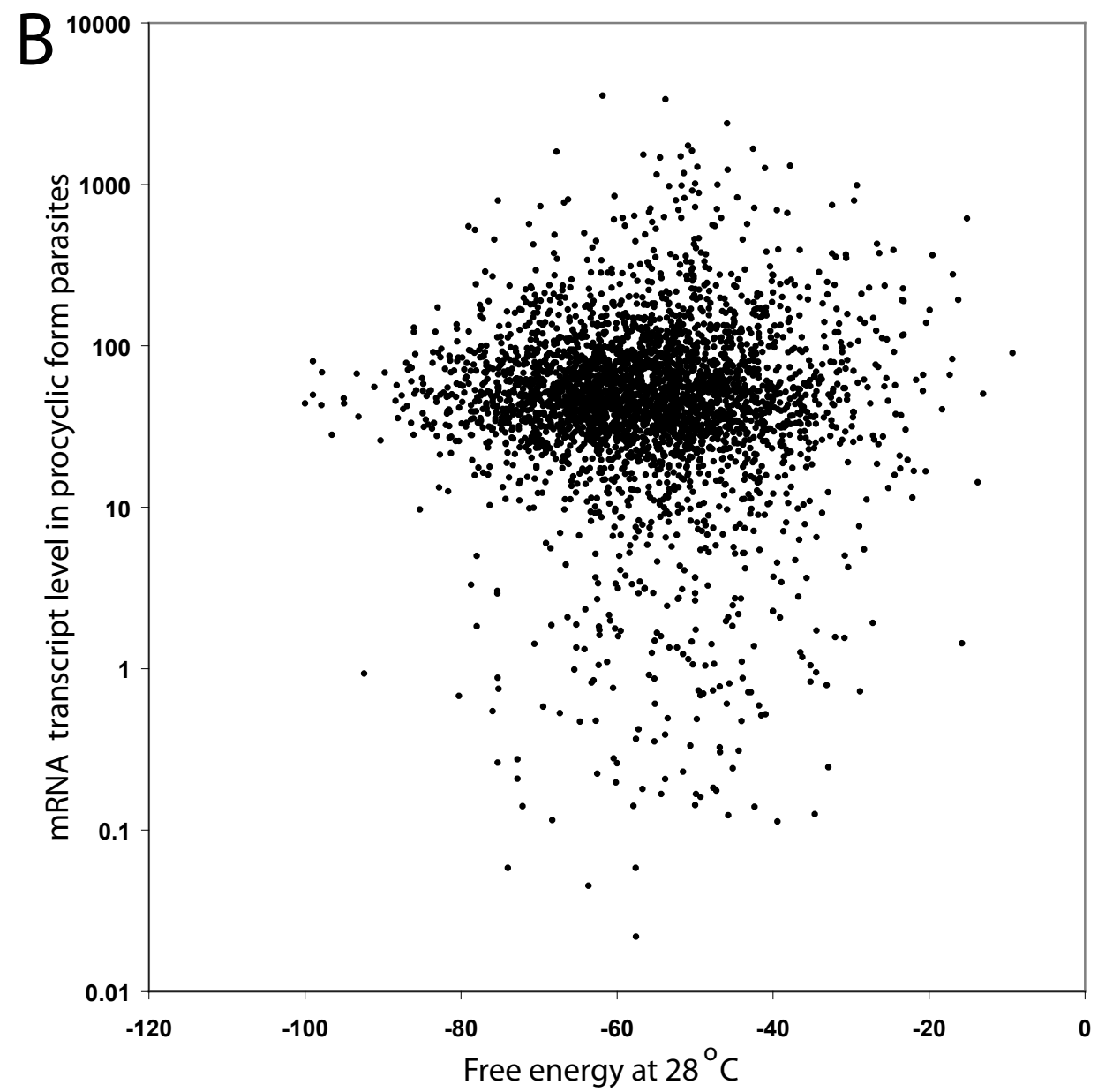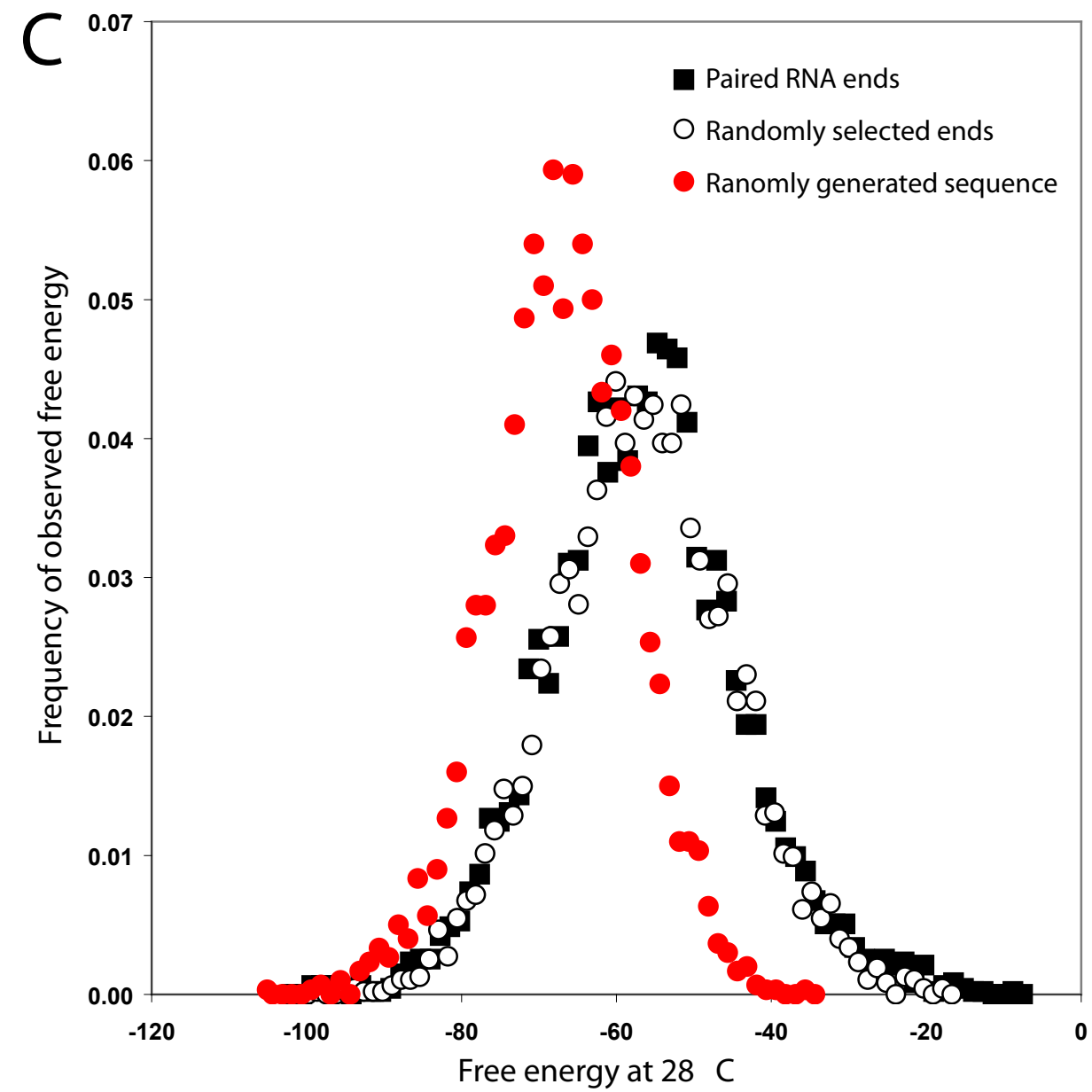

Supplement: File S8 — The free energy of hybridisation between the DNA immediately encompassing the coding sequence. A) Correlation between transcript level in bloodstream form parasites and free energy of hybridisation at 37°C. B) Correlation between transcript level in procyclic form parasites and free energy of hybridisation at 28°C. C) Comparison of distribution of free energies between paired ends (white circles) and random ends (black boxes) and randomly generated sequence (red circles). (PDF) [file pone.0025666.s008.pdf]
